# The contribution of comprehensive geriatric assessment to primary care physicians

**DOI:** 10.1186/2045-4015-3-44

**Published:** 2014-12-18

**Authors:** Shelley A Sternberg, Netta Bentur

**Affiliations:** Maccabi Healthcare Services, Azrieli Center, Beit Elazar, 1 Arar Street, Modiin, Israel; Myers-JDC-Brookdale institute, POB 3886, Jerusalem, 91037 Israel

## Abstract

**Background:**

To provide quality care to the growing number of older patients, primary care physicians (PCPs) will require support from geriatric specialists. Multidisciplinary comprehensive geriatric assessment (CGA) has been found to improve outcomes in older people. This study explored the contribution of CGA to the management of older patients by their PCPs; PCP attitudes to CGA; and PCP satisfaction with CGA.

**Methods:**

Two hundred PCPs in an Israeli Preferred Provider Organization were interviewed as part of an evaluative study of the contribution of a national outpatient CGA program to older patients, their families and physicians.

**Results:**

The main reasons for referral to CGA were cognitive impairment and rapid functional decline. Three domains described the contribution of CGA to PCPs: medical treatment, support in counseling patients, and treatment of cognitive impairment. About 69% of PCPs definitely agreed that CGA more fully addressed the physical, mental and social needs of patients than other consultative clinics. About half were very satisfied with the CGA staff’s attitudes to patients, their families and to the PCP.

**Conclusions:**

CGA contributed significantly to the care provided to older patients by PCPs. The expansion of CGA services deserves consideration.

## Background

As demographics shift to an aging population, primary-care physicians (PCPs) increasingly treat people with multiple chronic conditions and geriatric symptoms [1]. The quality of care provided to these vulnerable elders was assessed by Askari et al. in a systematic review of studies relying on a validated set of quality indicators [2]. The findings showed that the quality of primary care was low for chronic diseases and geriatric syndromes such as osteoarthritis, dementia, depression, falls, urinary incontinence, and end-of-life provisions. Future PCPs, to provide quality care to their older patients, will require more geriatric training and support from geriatric specialists [3–5].

The core of specialist care for older patients is the comprehensive geriatric assessment (CGA) [6]. This interdisciplinary approach rests on a geriatric physician, nurse and social worker at the very least. The team evaluates the patient, develops a program of care for the PCP and family, and follows up to assist with implementation.

CGA outcomes have been studied in many randomized control trials. Most of the research has found a high level of patient and family satisfaction, a decrease in the rate of functional decline, and an increase in the use of auxiliary services such as physiotherapy and social work. As a result, it has been recommended that CGA be standard practice in the care of older people [6].

Few of these studies, however, have looked at the contribution of CGA to the care provided by PCPs. In a study assessing physician and patient compliance with the recommendations of a community-based CGA program, Shah et al. found that physicians implemented 70% of the CGA recommendations for the management of falls, depression, urinary incontinence and functional impairment [7]. Aminzadeh, in a systematic review, and Press, in an Israeli study, found that physician adherence to CGA recommendations was enhanced by good geriatrician-PCP communication, limiting the number of recommendations, and empowering patients [8, 9]. A study from Thailand reported high PCP satisfaction with the CGA’s holistic approach [10]. All these studies point to the receptiveness of PCPs to CGA recommendations. But they do not explore the CGA’s specific contributions to the care given by PCPs or indicate which physicians are most likely to benefit.

Accordingly, the objectives of our study were to explore the contribution of CGA to the management of outpatients by their PCPs; the PCP attitudes to CGA; PCP satisfaction with CGA; and the impact of particular physician characteristics on the outcomes of care.

## Methods

We conducted a cross-sectional survey of PCPs in Maccabi Healthcare Services (MHS), the second largest Preferred-Provider Organization in Israel. MHS has a nationwide network of more than 3,000 physicians offering healthcare to 1.8 million members (24% of the country’s population). The survey of physicians was part of a study evaluating the contribution of a national CGA program to outpatients, families and PCPs [11]. It was approved by the Institutional Review Board (Helsinki committee) of MHS.

### Study population

All PCPs who had referred at least six patients for CGA in the year prior to the study (2008) were eligible to participate in the survey. This number was chosen as an indicator that the PCPs were familiar with the CGA program. Each of the five CGA clinics made available a list of eligible physicians in their area. The clinics included multidisciplinary teams of doctors, nurses, social workers, occupational therapists and, at times, physiotherapists. The teams served as consultants receiving referrals from family physicians. Each new patient was seen by everyone attending team meetings, where a treatment plan was developed. The assessment and summary meetings were spread over one or two visits and follow up was performed as needed, often on a six month to yearly basis. The PCPs and families received a letter from the computerized medical records system listing the recommendations along with additional information on community services.

### Study methods

All eligible physicians were sent a letter explaining the study and containing a self-administered questionnaire and a stamped return envelope.

The questionnaire contained open and closed questions:

1. "What are the main reasons for referral to CGA?" – an open-ended question with more than one answer permitted
2. The contribution of CGA to PCPs was examined by means of 17 closed statements assessing evaluation, diagnosis and treatment. The choice of response ranged from "very useful" to "useful", "not so useful" and "not useful at all" (Table  1).
3. A comparison of PCP attitudes to CGA and to other consultation clinics was assessed by nine statements (Table  2). For example: "The geriatric clinic addresses the physical, mental, social and family status of the patient more than do other consultation clinics". The choice of response ranged from "definitely agree" to "agree", "agree partially" and "do not agree at all".
4. PCP satisfaction with CGA was evaluated by responses to eight statements (Table  3). The choice of response ranged from "very satisfied" to "satisfied", "not so satisfied" and "not satisfied at all".

**Table 1:** **Characteristics of Maccabi Healthcare Services (MHS) primary-care physicians (PCPs) compared to the study population of PCPs**

	Total MHS PCPs (N = 1,500)	Study population of PCPs (N = 200)
**Average age**	50	51
**Gender (%)**		
Female	44	41
**Country of medical training (%)**		
Israel	34	34
Former Soviet Union	30	29
Other	36	37
**Specialty (%)**		
None	41	21
Internal medicine	31	31
Family medicine	28	48
**Employment status (%)**		
Salaried	23	27
Self-employed	77	63
Other (combination)	---	10

**Table 2:** **Contribution of CGA to primary-care physicians – factor analysis**

	Medical treatment	Support and counseling	Diagnosis and treatment of cognitive impairment
Identification of new diagnoses for referred patients	**0.725**	0.111	0.173
Diagnosis of depression	**0.753**	0.073	0.110
Provision of suitable medication for depression, change of medication for depression, etc.	**0.744**	0.157	0.084
Adjustment of medication for the illnesses and problems of the elderly patient	**0.621**	0.237	0.221
Coping with drug interactions	**0.686**	0.167	0.019
Coping with repeated falls, incontinence and other problems experienced by the patients	**0.516**	0.405	0.118
Recommendation for tests not yet carried out on the patient	**0.635**	0.261	0.000
Ability to provide the elderly patient with paramedical treatment such as physiotherapy, occupational therapy, etc.	**0.480**	0.432	0.111
Learning and acquiring experience of diagnosis and treatment of the elderly	**0.451**	0.298	0.439
Improved responsiveness of the patient to taking the medication s/he needs	0.396	**0.630**	0.022
Ability to counsel and/or influence the patient to receive home help or supervision	0.261	**0.740**	-0.002
Ability to counsel and/or influence family members to provide the elderly patient with help or supervision, social services, a foreign care worker, etc.	0.259	**0.746**	-0.024
Greater satisfaction of the patient and his/her family that you are doing all you can for him/her	0.117	**0.631**	0.351
To reassure and support the patient and his/her family	0.014	**0.698**	0.326
Confirmation of the diagnosis you have already made	-0.144	0.208	**0.587**
Diagnosis of cognitive decline, dementia, Alzheimer’s	0.201	0.059	**0.686**
Medication for cognitive decline, dementia, Alzheimer’s	0.265	-0.017	**0.657**

Values in bold reflect statements included in the specific domain.

**Table 3:** **Attitudes of primary-care physicians to CGA, by specialty ("definitely agree")**

	Total (%)	No specialization	Family medicine	Internal medicine
The geriatric clinic relates to the physical, mental, social and family status of the patient more than other consultation clinics do	**69**	70	71	62
The medication recommendations given by the geriatric clinic take into account the patient’s general condition more so than those given by other consultation clinics*	**50**	54	59	33
The recommendations I get from the geriatric clinic are more detailed than those I get from other consultation clinics*	**48**	46	55	39
With the help I get from the geriatric clinic, I learn more about diagnosing and treating the elderly than I do with the help of other clinics*	**41**	49	44	31
The recommendations given to the patients and their families at the geriatric clinic improve the quality of life of the elderly patients	**40**	49	40	35
The geriatric clinic is more considerate of the patient’s wishes than other clinics	**24**	32	24	18
My communication with the geriatric clinic is better than with other clinics*	**20**	22	26	9
The patients are more willing to comply with the recommendations of the geriatric clinic than of other clinics*	**15**	22	15	9
The geriatric clinics recommend additional testing more so than other consultation clinics	**12**	27	11	4

*Statistically significant for GPs/family physicians and specialists in internal medicine vs. other specialties (P < 0.05).

Values in bold reflect statements included in the specific domain.

### Statistical analysis

The data were analyzed with SPSS-18 software. Physician characteristics (age, sex, conditions of employment and specialization) were described using means (for age) and frequency distribution for categorical variables. Factor analysis using orthogonal varimax rotation was used (with Engle value more than 1) to derive underlying factors from the 17 statements describing the contribution of CGA to PCPs. To test the internal consistency of each of the domains in the factor analysis, Cronbach’s alpha was calculated. The association between physician characteristics and each domain was examined via multivariate linear regression. Finally, PCP attitudes to, and satisfaction with, CGA were described using frequency distributions. The association between physician characteristics and their attitudes to, and satisfaction with, CGA was analyzed using chi square methodology.

## Results

### Physician characteristics

In total, 360 physician names were provided by the five CGA clinics and 200 (56%) physicians agreed to participate. The main reasons for non-participation were lack of time (22%) and lack of response after three reminders (22%). Sixty percent of the PCPs were male with a mean age of 51 ± 8. Almost half were specialists in family medicine (48%), 31% were internists, and 21% were general practitioners with no specialization. Most (85%) worked only as PCPs; the rest worked additionally in hospitals or in medical administration. Two-thirds were self-employed while the remainder were MHS salaried employees.

As the questionnaire was anonymous, we were unable to compare the characteristics of participant and non-participant physicians. We could, however, compare our sample of participant physicians with all MHS PCPs and found them similar except for a lower proportion of general physicians and a higher proportion of family physicians in the sample (Table 1).

### Reasons for CGA referral

The PCPs cited more than one reason for referral. An identical percentage, 55%, cited cognitive impairment or rapid functional decline as the main reason for referral. Some 10% mentioned polypharmacy, depression, the need for placement in a nursing home or receiving government assistance for personal care. Only a few referred to incontinence, pain or sleeping problems.

### The CGA contribution to PCPs

Most physicians (76%) termed CGA "very useful" for the diagnosis and treatment of cognitive decline and dementia; 58% – for confirming diagnoses; 44% – in supporting families and patients, and reassuring them that the PCPs were doing everything possible; and 25% – for medication management.

An exploratory factor analysis of responses to the 17 statements yielded three domains or factors. Nine items were grouped as the first domain, describing the contribution of CGA to medical treatment (Cronbach’s alpha = 0.87). Five items were grouped as a second domain, describing the contribution of CGA to supporting PCPs in counseling patients (Cronbach’s alpha = 0.82). And three items were grouped as a domain describing the contribution of CGA to the diagnosis and treatment of cognitive impairment (Cronbach’s alpha = 0.48) Table 2 outlines the loading assigned to each one of the 17 statements. Values in bold were included in each of the three domains.

To determine the relationship between *physician characteristics* and each of the three domains, we conducted three multivariate linear regression analyses. Each domain was entered as a continuous dependent variable while the physician characteristics served as independent variables. The analyses revealed that CGA contributed significantly more to: the medical treatment domain of general and family physicians than of internists, and to salaried more than self employed physicians when controlling for age and sex; the counseling domain of salaried more than self-employed physicians when controlling for age, sex and specialization; the cognitive impairment domain of younger more than older physicians when controlling for sex, specialization and employment status.

### PCP attitudes to the CGA

Comparing CGA with other *consultative clinics* on the basis of statements with which the PCPs definitely agreed: about 69% responded that CGA more fully addressed the physical, mental and social needs of patients; about half responded that CGA addressed medication management better than other services; about 40% responded that CGA was more useful for diagnosis and treatment, and that CGA recommendations to patients and families improved quality of life. General physicians and family doctors were more likely to have positive attitudes to CGA than internists and other specialists (p < 0.05). (Table 3) Age, sex and employment status had no effect on PCP attitudes to the CGA.

### PCP satisfaction with CGA

About half of the PCPs were "very satisfied" with the professionalism of the CGA clinic staff and their attitudes to the patients and families; and about a third – with the quality of their communication with CGA staff, the diagnoses and recommendations, and with the guidance and counseling provided to family members on issues important to the well-being of older patients. By contrast, only 15% were "very satisfied" with the waiting time for an appointment at the CGA clinic. Significantly more family and general physicians than internists were "very satisfied" with the attitude of the CGA staff to them and the quality of communication (Table 4).

**Table 4:** **Satisfaction of primary-care physicians with CGA, by specialty ("very satisfied")**

	Total (%)	General medicine	Family medicine	Internal medicine
The professional level of the staff at the clinic	**54**	55	59	46
The attitude of the staff at the clinics to the patients and members of their families	**49**	53	52	40
The attitude of the physicians and staff at the clinics towards you personally*	**46**	56	50	31
Your communication with the physicians and staff at the clinics and your ability to talk with them when necessary*	**36**	48	39	22
Their diagnoses and their recommendations for medication, treatment, and social assistance services	**36**	32	42	28
The guidance to family members regarding the Community Long-term Care Insurance Law, supervision, safety in the home, and other issues that are important to the well-being of the elderly	**36**	39	40	29
The recommendations and guidance they give to the elderly and their families regarding functioning, use of social services (e.g., the Community Long-term Care Insurance Law), and recreational activities	**29**	24	34	24
Waiting time for appointments	**15**	16	15	13

*Statistically significant for GPs/family physicians and specialists in internal medicine vs. other specialties (P < 0.05).

Values in bold reflect statements included in the specific domain.

## Discussion

Our cross-sectional survey of PCPs showed that outpatient CGA contributed significantly to the care of patients with cognitive decline and dementia, the management of overall medical care, and the counseling and reassurance of patients and families. Most PCPs felt that CGA more fully addressed the physical, mental and social needs of older people than other consultative clinics. More than half of those surveyed were very satisfied with the professional standards of, and relationships with, staff at CGA clinics. In some cases, family physicians and general physicians benefited more than internists from CGA and viewed CGA clinics more favorably.

Our findings are consistent with a study from Thailand that reported high PCP satisfaction with the CGA’s holistic approach to care [10]. According to Summer et al. in a review article, CGA supports PCPs in their therapeutic relationship with patients. This finding was supported by our study [11].

Little information exists on PCP characteristics and their impact on the care of older patients. In a large secondary data analysis, Pham et al. showed that PCPs who were internists rather than family or general practitioners were more likely to deliver preventive services to their elderly Medicare beneficiaries [12]. By contrast, our study has shown that general physicians and family medicine specialists were more positive about CGA than internists. This difference in the impact of the PCP specialty on care provided to older patients may be due to the different services described; i.e., preventive care vs. CGA, or it may reflect particular characteristics of the US vs. Israeli healthcare systems. In addition, this difference across specialties might be due to the degree of exposure to geriatrics that physicians had in training, but this information was not available to us. In future studies it might be worthwhile to include this variable.

One of the limitations of our study is the 56% physician response rate. Although this is a recognized limitation of physician surveys, it may have biased the results [13]. In addition, we evaluated CGA contributions to PCPs solely on the basis of physician interviews, not by quantifiable endpoints such as the number of recommendations carried out, changes in medication etc. Physicians interviewed were those who regularly refer to CGA and not physicians who do not refer to CGA. Selection bias might have been introduced because physicians included in the study were by definition those that had a minimum of 6 referrals to CGA thus they actually represented physicians who had an awareness of the application of CGA to the care of their patients. Another issue worthy of further exploration in future studies is the relatively relative low correlation of the three items in the diagnosis and treatment of cognitive impairment domain of the factor analysis describing the contribution of CGA to the PCP.

## Conclusion

In conclusion, outpatient CGA contributed significantly to the care provided by PCPs. The implementation and expansion of CGA deserves consideration. Referrals from PCP to CGA for assessment and management of cognitive decline should be encouraged. In addition, since the majority of PCPs did not refer to CGA despite its potential significant contribution, standardized criteria for CGA referral should be developed and disseminated.
